# Objectively-assessed physical activity and weight change in young adults: a randomized controlled trial

**DOI:** 10.1186/s12966-017-0620-x

**Published:** 2017-12-04

**Authors:** Jessica L. Unick, Wei Lang, Samantha E. Williams, Dale S. Bond, Caitlin M. Egan, Mark A. Espeland, Rena R. Wing, Deborah F. Tate, SNAP Research Group, Rena R. Wing, Erica Ferguson, Kristen Annis, Ryan Busha, Isabella Cassell, Eva Chen, Pamela Coward, Jose DaCruz, Caitlin Egan, Brittany James, Marie Kearns, Angelica McHugh, Kevin O’Leary, Kathy Palmer, Deborah Ranslow-Robles, Kathryn Story, Jennifer Trautvetter, Jessica Unick, Samantha Williams, Carolyn Wunsch, Annajane Yolken, Deborah Tate, Karen E. Hatley, Candace Alick, Loneke Blackman, Rachel Bordogna, Melissa Crane, Molly Diamond, Kristen Polzien, Keneisha Quick, Brooke Tompkins Nezami, Carmina Valle, Cora E. Lewis, Amy Gorin, Jessica G. LaRose, Mark A. Espeland, Letitia H. Perdue, Judy L. Bahnson, Wei Lang, Cheryl Bentley, Patty Davis, Katelyn Garcia, Leah P. Griffin, Lea Harvin, Rebecca H. Neiberg, Julia Robertson, Santica M. Marcovina, Jessica Hurting, Vinod Gaur, S. Sonia Arteaga, Catherine Loria

**Affiliations:** 1 Brown University and the Miriam Hospital’s Weight Control and Diabetes Research Center, 196 Richmond Street, Providence, RI 02903 USA; 2 Wake Forest School of Medicine, Winston-Salem, NC USA; 3 University of North Carolina, Gillings School of Global Public Health, Chapel Hill, NC USA

**Keywords:** Weight gain, Young adulthood, Exercise, Physical activity, Body weight

## Abstract

**Background:**

Reductions in physical activity (PA) are common throughout young adulthood and low PA is associated with weight gain. The SNAP Trial previously reported that two self-regulation approaches to weight gain prevention reduced weight gain over a 2-year period in 18–35 year olds. Presented here are secondary analyses examining changes in PA and the relationship between PA and weight change over 2 years.

**Methods:**

599 young adults (age: 27.4 ± 4.4 yrs.; BMI: 25.4 ± 2.6 kg/m^2^) were randomly assigned to 1 of 3 treatment arms: Small Changes (reduce calorie intake by 100 kcals/day & add 2000 steps/day), Large Changes (lose 2.3–4.5 kg initially & increase PA to ≥250 min/wk), or Self-guided (control condition). Small and Large Changes received 10, face-to-face group sessions (months 1–4), and two 4-week refresher courses each subsequent year. Body weight and PA were objectively-measured at baseline, 4 months, 1 and 2 years. Daily steps and bout-related moderate-to-vigorous intensity PA (MVPA: ≥3 METs, ≥10-min bouts) was calculated.

**Results:**

Changes in bout-related MVPA and daily steps did not differ among treatment groups over the 2-year period (*p*’s > 0.16). Collapsed across groups, participants gaining >1 lb. (*n* = 187; 39.6%) had smaller changes in bout-related MVPA at 4 months, 1 and 2 years relative to those maintaining or losing weight (≤1 lb. weight gain; *n* = 282, 60.4%, *p*’s < 0.05). Averaged across time points, this difference equated to 47.8 min/week. Those gaining and not gaining >1 lb. did not differ on daily steps (*p*’s > 0.10). Among participants engaging in ≥250 min/wk. of MVPA at 2 years (*n* = 181), 30% gained >1 lb. from baseline to 2 years, which was not different from those engaging in 150–250 min/wk. (*n* = 87; 36%; *p* = 0.40), but this percentage was significantly lower when compared to those engaging in <150 min/wk. (*n* = 176; 49%; *p* < 0.001).

**Conclusions:**

On average, PA differences were not observed between young adults assigned to small or large changes self-regulation interventions to prevent weight gain. Regardless of group assignment, higher levels of MVPA were associated with better weight gain prevention over 2 years. Our data suggest that achieving >150 min/week of MVPA is needed for weight gain prevention and that increasing MVPA, rather than steps, should be targeted.

**Trial registration:**

www.clinicaltrials.gov (NCT01183689). Registered Aug 13, 2010.

**Electronic supplementary material:**

The online version of this article (10.1186/s12966-017-0620-x) contains supplementary material, which is available to authorized users.

## Background

Previous literature demonstrates that young adults (ages 18–35) experience a faster rate of weight gain than other age groups, gaining an average of 1 lb./year [1, 2]. This is of concern given that rapid weight gain among young adults has been linked to elevated cardiovascular disease risk factors and other adverse health outcomes [3]. Of additional concern is that a higher BMI earlier in life is associated with having a higher BMI later in life [4, 5]. Thus preventing weight gain throughout young adulthood could have significant public health implications.

Young adulthood is also marked by reductions in physical activity (PA), which typically begin during adolescence and continue throughout young adulthood. This is a highly transitional period, often characterized by significant life events (e.g., starting a new job, getting married, or having children); thus it is possible that these major life transitions may be a contributing factor to the observed decrease in PA [6, 7] and subsequent weight gain [8, 9]. Currently, the role of PA in weight gain prevention among young adults is not currently understood. According to the American College of Sports Medicine, there is adequate evidence that 150–250 min/week of moderate-to-vigorous intensity PA (MVPA) is sufficient to prevent significant weight gain in the general population [10]. Given that weight gain is most common in young adults, it is unclear whether this magnitude of PA is also sufficient for preventing weight gain in this age group.

To date, the body of literature related to PA and weight gain among young adults is limited. The majority of studies have been cross-sectional (i.e., comparing PA patterns in young adults to other age groups) or longitudinal (i.e., examining changes in PA over time within a cohort of individuals) [7, 11–13] and few intervention studies have been conducted. Thus, little is known about how PA and body weight change within the context of a lifestyle intervention or whether changes in PA are related to changes in body weight among young adults. An additional concern is that the majority of PA studies have utilized self-reported measures of PA, which are prone to participant biases due to social desirability or imprecise recall [14]; thus it is unclear whether similar findings would be observed when objective PA measures were used. Given these significant gaps in the literature, coupled with the high risk nature of weight gain among this demographic group, it is evident that well-designed intervention studies aimed at increasing PA and preventing weight gain are needed. Studies such as these are critical for determining whether objectively-assessed PA is improved as a result of lifestyle intervention in young adults and whether changes in PA are related to changes in body weight.

The Study of Novel Approaches to Weight Gain Prevention (SNAP) trial overcomes some of these previous limitations and provides an excellent opportunity to examine the relationship between objectively-assessed PA and weight change over a 2-year period among young adults interested in weight gain prevention. The SNAP trial compared two self-regulation weight gain prevention programs (e.g., ‘Large Changes’ and ‘Small Changes’) to a minimal contact control condition (i.e., ‘Self-Guided’) over a two-year period. The primary aim was to examine changes in weight across the three intervention arms, and these data have been previously published [15]. The current analyses focus on whether there was a differential effect of intervention arm on objectively-assessed PA over the 2-year period and whether compliance to the PA recommendations differed by group. Secondary aims were to examine the impact of baseline PA on changes in PA over time and to determine whether there is a relationship between PA and weight gain prevention over the intervention period.

## Methods

### Participants

Young adults (*n* = 599) interested in weight gain prevention enrolled in the SNAP trial between August 2010 and February 2012. Participants were recruited primarily by mass mailings (38%) and emails (23%), using text that sought individuals who were concerned about gaining weight over time [16]. Participants were normal weight (BMI: 21 to <25 kg/m^2^) or overweight (BMI: 25 to 30 kg/m^2^), between the ages of 18 and 35, English speaking, and had no medical conditions that would limit their ability to make dietary or PA changes. Eligible individuals were required to pass screening and baseline assessment visits. Full exclusion criteria have been previously reported [17].

### Design

The SNAP trial examined two novel interventions for weight gain prevention compared to a control condition in young adults. Participants were randomized to one of three treatment arms: Small Changes (SC), Large Changes (LC) or Self-Guided (SG), which served as the control condition. Participants in LC and SC received a lifestyle intervention for 4 months (10 face-to-face group meetings), followed each year by two four-week refresher courses delivered primarily via the Internet. A detailed description of these treatment groups has been reported previously [17] and intervention components are also summarized below. All groups completed assessment visits at baseline, 4 months, 1 year, and 2 years post-treatment. Informed consent was obtained from all participants, and procedures were performed in accordance with The Miriam Hospital’s (Providence, RI) and University of North Carolina (Chapel Hill, NC) Institutional Review Boards.

### Randomization

Randomization assignment used variable block lengths, was stratified by clinical site, sex, and ethnicity (non-Hispanic white/other), and was implemented through a web-based data management system.

### Treatment groups

#### Intervention components common to both large changes and small changes

Participants randomized to LC or SC attended weekly in-person, group-based sessions for 8 weeks, followed by 2 monthly sessions, and were offered two 4-week, online refresher courses for each successive year of the study. While dietary and PA recommendations differed between SC and LC, both treatment groups emphasized daily self-weighing and participants were instructed to record their weight daily throughout the course of the study. Self-regulation techniques, such as detecting small changes in weight as soon as they occur and implementing problem solving and behavioral strategies to counteract the weight gain, were used in both groups.

#### Small changes

Participants randomized to SC were instructed to make daily, small changes in diet and PA in order to prevent weight gain. Dietary recommendations focused on reducing calorie intake by 100 cal per day through ‘small’ behavior modifications, such as reducing portion sizes or selecting lower calorie alternatives. Further, SC participants were given pedometers and instructed to increase daily steps by 2000 steps/day above their baseline level (equivalent to 1 mile of walking) through changes in lifestyle activities (e.g., parking further from the store or using the stairs). Participants were given a monthly chart to record their daily weight, steps, and whether they made any small changes to their diet. This was completed daily during the first 16 weeks and during refresher courses. These were reviewed by interventionists and feedback was provided.

#### Large changes

Participants randomized to LC were instructed to make larger changes to their diet and PA to create a 5 to 10 pound buffer against future weight gain within the first 4 months [18]. Participants were instructed to reduce calorie intake by 500–1000 kcals/day (depending upon initial body weight) and increase PA gradually to ≥250 min/week of MVPA. Once this ‘buffer’ was created, participants were instructed to gradually increase calorie intake to maintain their reduced weight and to maintain this high level of PA throughout the remainder of the study. If at any point a participant’s weight exceeded their baseline weight, it was recommended that they return to their initial calorie intake and recreate another 5–10 lb. buffer. Participants were instructed to record their weight, diet, and minutes of PA daily. These diaries were reviewed by an interventionist and feedback was provided.

#### Self-guided (control condition)

Participants in the control condition attended one in-person group session and were provided with general information on weight gain in young adults, which included basic guidelines for self-weighing and a brief overview of both SC and LC approaches. They were then encouraged to select the approach that would work best for them and apply these strategies over the course of the study. Participants were sent quarterly newsletters via postal mail and were provided with links to internet resources via a study website but received no additional contact from intervention staff.

### Assessments

All assessments were completed by masked staff members, who were centrally trained and certified.

#### Anthropometric

Height and weight were measured at baseline, 4 months, 1 year, and 2 years. Height was measured using a wall-mounted stadiometer and weight was measured in light clothing without shoes on a calibrated scale.

#### Dietary intake

Dietary intake was assessed at baseline, 4 months, and 2 years using the Block Food Frequency Questionnaire [19]. Dietary comparisons between treatment arms will be reported in a separate manuscript. However, total daily caloric intake and percentage of total calories from dietary fat were controlled for in all analyses which assessed the relationship between PA and weight change.

#### Physical activity

Physical activity was assessed using the previously validated Sensewear Armband (SWA, BodyMedia, Pittsburgh, PA) [20–22]. The SWA is worn on the back of the upper arm and assesses PA using a biaxial accelerometer and a combination of heat sensors. Participants were instructed to wear the device during all waking hours (except while bathing or swimming) for 7 consecutive days at each assessment time point and data were considered to be ‘valid’ if wear time was ≥8 h on ≥4 days. Proprietary algorithms produced minute-by-minute estimates of energy expenditure (expressed as metabolic equivalents or METs) using the Sensewear Professional Software (Version 7.0). These MET values were used to calculate ‘bout-related’ moderate-to-vigorous intensity PA (MVPA), which includes activities ≥3.0 METs and ≥10 min in duration. Further the SWA provided estimates of daily steps. The proportion of participants meeting the national PA recommendation for improved health (e.g., ≥150 min/week of bout-related MVPA [23]), weight control (≥250 min/week of bout-related MVPA [10]), and daily steps (10,000 steps/day [24]) were also examined.

### Statistical analyses

Data were analyzed between May 2016 and February 2017. Statistical analyses were performed using SAS (version 9.4). The type I error rate was fixed at 0.05 (two-tailed). Descriptive statistics included mean and standard deviation (SD) or median and interquartile range (IQR) for continuous measures, depending on the normality of distribution, and count and percentage for categorical variables.

To examine differences among the three treatment groups in the changes of daily steps, bout-related MVPA, and body weight, separate mixed effects models were fit to the changes from baseline in these outcomes, with three time points (4 months, 1- and 2- years). Each model was adjusted for the following covariates: clinic, gender, race (White vs. non White), and baseline value of the corresponding outcome. Both mixed effects models for the changes of daily steps and bout-related MVPA also adjusted for the time-varying covariate of armband wear time. Significance of treatment group, time, and treatment group by time interaction effects were assessed in these models using the unstructured dependence structure. Results from the mixed model analyses were presented as the least square mean with 95% confidence interval.

Dichotomous outcomes were defined over time for meeting ≥250 min/week of bout-related MVPA and for increasing daily steps by ≥2000 steps/day. Subsequently, these dichotomous outcomes were modeled using the generalized estimating equations (GEE) approach, adjusting for covariates of clinic, gender, race (White vs. non White), armband wear time, and baseline value of either bout-related MVPA or daily steps.

The Wilcoxon rank sum test was used to test the differences in changes in daily steps and bout-related MVPA between two groups: those who gained >1 lb. and those who gained ≤1 lb. or lost weight from baseline to 2 years. A binary indicator variable (= 1 if gained >1 lb. from baseline to 2 years, =0 otherwise) was added to the mixed effects models described above. This model also adjusted for dietary intake covariates (total daily caloric intake and percentage of total calories from dietary fat). Regression coefficient, standard error, and *p*-value for the binary indicator are presented in Table 3.

## Results

### Participants

Subject characteristics have been described in detail previously [17]. At baseline, four participants failed to meet the minimal armband wear time criteria, resulting in analysis sample of *n* = 595. Participants were predominately white (73%) and female (78%), 27.7 ± 4.4 years of age, with a mean BMI of 25.4 ± 2.6 kg/m^2^. Participants with weight data and those meeting the minimal armband wear time threshold at each time point were included in the analyses (baseline: 99%, 4 months: 92%, 1 year: 79%, 2 years: 76% of participants) and retention rates did not differ by treatment arm at any time point (*p*’s > 0.08, see Appendix 1 Consort diagram and Additional file 1: checklist). Compliance to wearing the armband was excellent at baseline (7.1 ± 0.9 days for 14.1 ± 1.5 h/day) and remained high at 4 months (6.6 ± 1.3 days for 13.7 ± 1.8 h/day), 1 year (6.6 ± 1.4 days for 13.7 ± 1.8 h/day) and 2 years (6.6 ± 1.4 days for 13.6 ± 1.6 h/day). On average, study participants were highly active at baseline with 60.2% achieving ≥150 min/week of bout-related MVPA, 40.8% achieving ≥250 min/week of bout-related MVPA, and 28.4% averaging the national recommendation for daily steps (≥10,000 steps/day). Attendance at face-to-face intervention meetings did not differ between SC (86.0%) and LC (87.4%).

### Change in physical activity throughout the intervention by treatment arm

The primary aim was to examine whether there was a differential effect of treatment arm on objectively-assessed PA over the 2-year period. The group by time interaction effect was not significant, indicating that the pattern of change over time for both daily steps and bout-related MVPA did not differ between treatment arms (Table 1). Further, in models adjusting for demographic variables, 4-month, 1-year, and 2-year change in daily steps and bout-related MVPA did not significantly differ across treatment groups. However, there was a significant time effect such that, when collapsed across treatment arms, changes in daily steps at month 4 was significantly greater than changes at year 1 (*p* = 0.015) and year 2 (*p* = 0.006), while changes in weekly bout-related MVPA was significantly greater at 4 months compared to year 1 (*p* = 0.013).

**Table 1:** Physical activity over time stratified by treatment arm

		Assessment Periods				*p*-values		
	Treatment Group	Baseline Mean ± Std dev Median (IQR)	BL – 4 mo change^a^	BL – 1 year change^a^	BL – 2 year change^a^	Treatment Group	Time	Treatment Group x Time
Daily Steps	Control	8866 ± 3146 8267 (6674, 10,872)	342 (−42, 727)	−135 (−537, 268)	−179 (−635, 277)	0.7434	0.0118*	0.2976
	Small Change	8450 ± 2997 8032 (6337, 9933)	139 (−253, 531)	221 (−198, 641)	−71 (−534, 392)			
	Large Change	8622 ± 3051 8383 (6366, 10,513)	260 (−138, 658)	−258 (−668, 153)	−205 (−668, 259)			
Bout-related MVPA (min/week)	Control	260.4 ± 254.7 177.6 (88.0, 352.3)	54.5 (20.1, 89.0)	12.2 (−26.4, 50.8)	20.8 (−26.7, 68.3)	0.6604	0.0442*	0.1569
	Small Change	253.4 ± 246.6 180.0 (85.8, 329.0)	37.8 (2.8, 72.8)	43.9 (3.8, 84.1)	24.0 (−23.8, 71.8)			
	Large Change	276.5 ± 237.8 211.4 (84.0, 400.0)	66.5 (30.8, 102.1)	11.2 (−28.0, 50.4)	63.6 (15.4111.7)			
Weight (kg)	Control	71.4 ± 10.2 70.7 (63.6, 78.1)	−0.8 (−1.2, −0.3)	−0.4 (−1.0, 0.3)	0.7 (0.0, 1.4)	<0.0001**	<0.0001**	0.0160*
	Small Change	71.9 ± 11.0 70.0 (63.6, 79.5)	−1.6 (−2.0, −1.1)	−1.4 (−2.0,-0.7)	−1.0 (−1.8, −0.3)			
	Large Change	70.8 ± 11.0 69.2 (63.4, 76.4)	−3.5 (−3.9, −3.0)	−2.5 (−3.1, −1.9)	−1.6 (−2.3, −0.8)			

^a^Least Square Means (95% confidence interval); Steps and MVPA models adjust for clinic, gender, race, armband wear time, and baseline value of outcome measure. Weight model adjusts for clinic, gender, race, and baseline weight. Adjusted change values are presented. Boldface indicates statistical significance (*p < 0.05, **p < 0.001)

Compliance to the intervention PA recommendations for LC (≥250 min/week of bout-related MVPA) and SC (increase steps by ≥2000 steps/day) was also assessed within treatment arms. The percentage of participants engaging in ≥250 min/week of bout-related MVPA did not change over the 2-year intervention period and did not differ between treatment groups (Table 2). Further, the percentage of participants increasing their steps by ≥2000 steps/day above baseline did not change over time or differ by treatment arm (Table 2). Finally, attendance at intervention meetings was not associated with the change in daily steps or change in MVPA at any time point (e.g., 1-year: SC: steps: *r* = 0.10, *p* = 0.24, MVPA: *r* = −0.05, *p* = 0.57; or 1-year LC steps: *r* = 0.09, *p* = 0.23, MVPA: 0.14, *p* = 0.07).

**Table 2:** Percentage of participants meeting intervention PA recommendations

		Assessment Periods				*p*-values		
	Treatment Group	Baseline	4 months	1 year	2 years	Treatment Group	Time	Treatment Group x Time
≥250 min/week of bout-related MVPA	Control	81 (40.3%)	83 (43.2%)	65 (40.0%)	63 (41.5%)	0.2949	0.4872	0.1460
	Small Change	74 (37.2%)	82 (45.6%)	67 (44.7%)	56 (37.6%)			
	Large Change	88 (45.1%)	98 (55.4%)	65 (40.9%)	71 (46.7%)			
Increase daily steps by ≥2000 steps/day	Control	–	32 (16.7%)	25 (15.3%)	31 (20.4%)	0.1859	0.5124	0.8087
	Small Change	–	44 (24.4%)	39 (26.0%)	34 (22.8%)			
	Large Change	–	40 (22.6%)	35 (22.0%)	32 (21.1%)			

N (%); Models adjusted for clinic, gender, race, armband wear time, and baseline value of outcome measure

### Effect of baseline physical activity on change in physical activity over time

Given that the mean PA levels at baseline were high (> 250 min/week), a secondary aim was to examine whether there was an effect of baseline PA on the change in PA over time and to determine whether this differed by treatment arm. After adjusting for gender, race, and armband wear time, baseline PA significantly predicted the 2-year change in PA. Those with lower bout-related MVPA at baseline had more favorable changes in bout-related MVPA across the 2-year period (β = −0.38, *p* < 0.001). For example, averaged across treatment arms, participants engaging in <250 min/week of bout-related MVPA at baseline (*n* = 352) increased MVPA at 4 months (*N* = 335, 97.3 ± 186.9 min/week), 1 year (*N* = 287, 72.8 ± 182.7 min/week), and 2 years (*N* = 274, 98.0 ± 251.3 min/week), while those engaging in ≥250 min/week at baseline (*n* = 243) had reductions in MVPA at each of these time points (4 mo: *N* = 228, −32.9 ± 295.4, 1 year: *N* = 208, −75.6 ± 340.9, 2 years: *N* = 200, −73.5 ± 444.8 min/week). Similarly, those with lower daily steps at baseline had more favorable changes in daily steps from baseline to 2 years (β = −0.42, *p* < 0.001). The effect of baseline PA on the change in PA over time was similar in the 3 treatment arms (daily steps: *p* = 0.59; MVPA: *p* = 0.29).

### Effect of physical activity on weight change over time

Another aim was to examine the relationship between PA and weight change across the intervention period. Although the SNAP primary outcome paper has already reported on changes in body weight across treatment arms [15], weight data for the current sample are shown in Table 1. We compared daily steps and bout-related MVPA among participants gaining >1 lb. from baseline to 2 years (*n* = 187) to those who lost weight or gained ≤1 lb. over this same time period (*n* = 285). Participants with a 2-year weight gain >1 lb. engaged in less bout-related MVPA at 4 months, 1 year and 2 years, compared to those not gaining >1 lb. over this time period (Table 3). Further mixed effects models, adjusting for demographic variables, treatment group, dietary intake, and baseline values, revealed the change in MVPA across all time points differed by an average of 47.8 min/week between those gaining >1 lb. and those who did not (*p* = 0.0125). There was no significant difference in daily steps at 4 months, 1 year, or 2 years between those gaining >1 lb. and those gaining ≤1 lb. at year 2 (*p*’s > 0.10).

**Table 3:** Physical activity levels based upon gaining or not gaining ≥1 lb

		Baseline to Month 4 change			Baseline to Year 1 change			Baseline to Year 2 change			Mixed effects model*	
		N	Mean ± std. dev Median (IQR)	*p*-value^+^	N	Mean ± std. dev Median (IQR)	*p*-value^+^	N	Mean ± std. dev Median (IQR)	*p*-value^+^	Beta coefficient (std err)	*p*-value
Change in daily steps												
Gained >1 lb. from baseline to Year 2	Yes	182	−49 ± 2964 −104 (−1436, 1588)	0.1253	158	−346 ± 2598 −40 (−1794, 1137)	0.3328	173	−568 ± 2947 −388 (−2480, 1224)	0.1015	−302 (201)	0.1346
	No	275	304 ± 2525 375 (−1017, 1654)		263	111 ± 2798 −37 (−1583, 1714)		271	27 ± 3073 −45 (−1837, 1875)			
Change in bout-related MVPA (min/week)												
gained >1 lb. from baseline to Year 2	Yes	182	14.2 ± 239.7 0.0 (−90.3, 112.0)	0.0189*	158	−37.9 ± 276.9 −20.2 (−122.8, 80.5)	0.0051**	173	−34.4 ± 313.3 −40.0 (−144.7,89.0)	0.0023**	−47.8 (19.1)	0.0125*
	No	275	49.8 ± 234.0 51.0 (−59.0, 186.4)		263	39.5 ± 279.2 25.9 (−90.0, 139.0)		271	59.2 ± 334.4 22.8 (−94.0,163.3)			

^+^ *p*-value obtained using the Wilcoxon Rank Sum Test. Mixed effects model examines the change in physical activity across all time points. Other variables included in this model are: clinic, gender, race, total daily caloric intake, percentage of calories consumed from dietary fat, armband wear time, baseline value of outcome measure, treatment group, and treatment group x time interaction. Boldface indicates statistical significance (*p < 0.05, **p < 0.01)

**Fig. 1:**
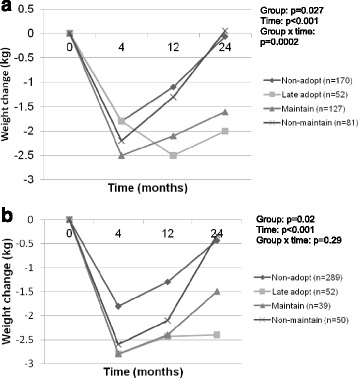
**a** Title: Categorization of participants based upon achievement of ≥250 min/week at 4 months and 2 years. **a** Legend: ‘Non-adopt’: <250 min/week at 4 months and 2 years; ‘Late adopt’: <250 min/week at 4 months, but ≥250 min/week at 2 years; ‘Maintain’: ≥250 min/week at 4 months and 2 years; ‘Non-maintain’: ≥250 min/week at 4 months but <250 min/week at 2 years. Model adjusts for clinic, gender, race, dietary intake, baseline weight, and baseline PA. **b** Title: Categorization of participants based upon achievement of ≥2000 steps/day above baseline at 4 months and 2 years. **b** Legend: Non- adopt: <2000 step increase above baseline at 4 months and 2 years; Late Adopt: <2000 step increase above baseline at 4 months but > = 2000 step increase at 2 years; Maintain: > = 2000 step increase above baseline steps at 4 months and 2 years; Non-maintain: > = 2000 step increase above baseline at 4 months and <2000 step increase above baseline at 2 years. Model adjusts for clinic, gender, race, dietary intake, baseline weight, and baseline steps

To further investigate the relationship between change in PA and change in weight, we used a categorical approach, examining how different patterns of PA change between 4 months (i.e., the end of the intensive intervention phase) to 2 years were related to weight change over this same time period. Participants were categorized into 1 of 4 PA groups based upon their achievement of ≥250 min/week of bout-related MVPA at 4 months and 2 years: 1) ‘Non-adopt’: <250 min/week at 4 months and 2 years, 2) ‘Late adopt’: <250 min/week at 4 months, but ≥250 min/week at 2 years, 3) ‘Maintain’: ≥250 min/week at 4 months and 2 years, and 4) ‘Non-maintain’: ≥250 min/week at 4 months but <250 min/week at 2 years (Fig. 1a). Similar categories were formed using the SC daily step goal, stratifying participants based upon whether they had a ≥ 2000 steps/day increase from baseline at both 4 months and 2 years (Fig. 1b). There was a significant group x time interaction effect for bout-related MVPA category on weight change over time (*p* = 0.0002). Independent of 4-month MVPA levels, participants engaging in <250 min/week at 2 years (‘Non-adopt’ and ‘Non-maintain’ groups) regained all of their weight from 4 months to 2 years, while those engaging in ≥250 min/week at Year 2 (‘Late adopt’ and ‘Maintain’ groups) had a mean weight loss of 1.5–2.0 kg at Year 2. There was not a significant group x time interaction effect when participants were categorized based upon steps.

Finally, we explored whether engagement in ≥250 min/week of bout-related MVPA (*n* = 181) was associated with more favorable changes in weight at 2 years, compared to 150 to <250 (*n* = 87) and 0–150 min/week (*n* = 176). Two-year weight change among participants engaging in ≥250 min/week (−1.83 ± 4.24 kg) was not significantly different from those engaging in 150 to <250 min/week (−0.78 ± 4.84 kg; *p* = 0.11) but was significantly greater than those engaging in 0–150 min/week (−0.06 ± 4.69 kg; *p* = 0.0001). Of note, <150 and 150 to <250 min/week groups were not significantly different from one another (p = 0.11). Further, 30.4% of participants engaging in ≥250 min/week gained >1 lb. at year 2, which was significantly lower than the 49.4% observed in the 0–150 min/week group (*p* = 0.0002), but not significantly different from those engaging in 150 to <250 min/week at year 2 (35.6%; *p* = 0.40). This percentage was also significantly higher in the 150 to <250 group, relative to the <150 min/week group (*p* = 0.03).

## Discussion

The SNAP trial examined the effectiveness of two different self-regulation approaches to weight gain prevention, relative to a control condition, in a large cohort of young adults. The primary outcome paper focused on changes in weight [15] – here we examine whether changes in objectively-assessed PA differed by treatment arm. Overall, the change in daily steps and bout-related MVPA over two years did not differ among SC, LC, or SG. When collapsed across treatment arms, the greatest improvement in PA was observed at 4 months. On average, participants increased bout-related MVPA by 50 min/week, which given the high PA levels observed at baseline, equated to over 300 min/week of bout-related MVPA at Month 4.

To date, few studies have examined changes in PA among young adults within the context of a lifestyle intervention and the SNAP trial was the first to examine this within a weight gain prevention trial. The IDEA study assessed the effect of a standard behavioral weight loss program for overweight and obese young adults on changes in objectively-assessed PA over 6 months [25]. Study participants were given an exercise goal of 300 min/week of MVPA. While IDEA participants were less active than SNAP participants at baseline (100 min/week vs. 263 min/week of bout-related MVPA), IDEA participants significantly increased bout-related MVPA to 215 min/week at 6 months. While the current study also reported increases in MVPA, although to a lesser degree than the IDEA study, SC and LC intervention approaches were no more effective at increasing MVPA than the SG group. Although we can’t say with certainty why the 3 groups did not differ on changes in PA, we hypothesize that this may be attributed to the fact that PA was only a small component of the SC or LC interventions. Specifically, only 1 of the 8 initial weekly intervention sessions in SC and LC focused on PA. Conversely, SG participants were provided with an overview of the principles of both the LC and SC approaches at one intervention session and encouraged to select whichever approach they felt would be most effective for them. Therefore, they were given the same general instructions for increasing PA as SC and LC, possibly explaining why similar changes in PA were observed between treatment groups. These findings suggest that for individuals enrolled in a program to prevent weight gain and who are taught that it is important to increase PA to achieve this goal, will do so, at least temporarily.

A secondary aim of this study was to examine the relationship between PA and weight change within the context of an intervention. While changes in PA were associated with changes in weight, this relationship did not differ by treatment group. We previously reported that a ≥ 1 lb. weight gain was associated with worsened cardiometabolic outcomes compared to weight loss or <1 lb. weight gain [26]. Given the clinical significance of gaining >1 lb., this paper examined whether there were differences in PA between these post-hoc weight groupings. Participants who lost weight or gained ≤1 lb. at year 2 had a 59 min/week increase in MVPA above baseline levels at year 2, while those participants who gained >1 lb. had a 34 min/week decrease in MVPA; differences in bout-related MVPA between these post-hoc weight groups were also observed at 4 months and 1 year. These results remained after adjusting for dietary intake. Interestingly, there was no difference in daily steps between those who gained >1 lb. and those who did not. This suggests that more structured exercise of at least moderate intensity may be more important than lifestyle activities for preventing weight gain; thus weight gain prevention programs should consider targeting changes in bout-related MVPA.

In addition to looking at differences in PA by post-hoc weight change groups, we also examined whether achievement of ≥250 min/week of MVPA was associated with weight change. While the percentage of participants achieving this threshold of PA at all time points did not differ by treatment group, when collapsed across treatment groups, achievement of ≥250 min/week at Year 2 was associated with improved 2-year weight outcomes when compared to those achieving <250 min/week. Moreover, this association persisted, regardless of bout-related MVPA at 4 months or dietary intake. However, follow-up analyses revealed that those achieving ≥250 min/week at year 2 lost approximately 1 kg (2.3 lbs) more than those engaging in 150 to <250 min/week of MVPA, which was not statistically significant. These data suggest that PA levels ≥150 min/week are also effective for preventing weight gain. This level of PA is consistent with recommendations from the American College of Sports Medicine for weight gain prevention, which state that there is sufficient evidence that 150–250 min/week of MVPA is sufficient to prevent weight gain greater than 3% in most adults [10]. The current findings confirm this recommendation in a sample of young adults, and expand upon this recommendation through the use of objective PA monitors, versus self-report measures of PA. Thus future weight gain prevention efforts in young adults should target ≥150 min/week of MVPA.

Unlike bout-related MVPA, achievement of ≥10,000 steps/day was not associated with weight change. While daily steps encompass both light intensity and MVPA, it appears that bout-related MVPA may be the greatest contributor for preventing weight gain. However, it should be noted that a greater percentage of SNAP participants achieved and maintained the MVPA threshold for PA than those who achieved and maintained ≥10,000 steps/day. Thus it is possible that achievement of a different threshold of daily steps may be more closely related to weight change.

Overall, SNAP participants were highly active at baseline – mean MVPA levels were >250 min/week and over 40% of participants met this threshold of PA. It is unclear whether this magnitude of PA is common for young adults of this BMI, or whether young adults enrolling in a weight gain prevention trial may be more likely to engage in higher levels of PA. Unfortunately, few studies have objectively-assessed MVPA among young adults. Overweight and obese young adults participating in a weight loss study (IDEA Study), engaged in >150 fewer min/week compared to SNAP participants at baseline [25]; however it is uncertain how much of this difference can be attributed to differences in BMI between study participants. Similarly in a population-based study, Tucker et al. reported that only 10.8% of young adults aged 20–29 engage in an ‘adequate’ amount of MVPA according to guidelines; however obese individuals were also included in these estimates [6]. Therefore, whether baseline PA among SNAP participants is ‘typical’ of young adults in this BMI range cannot be determined.

The final aim of this study was to examine the effect of baseline PA on change in PA over time. Baseline PA was a significant moderator of change in PA, with higher baseline PA associated with less favorable changes in PA over the intervention period. For example, participants in the current study who engaged in <250 min/week at baseline increased PA by approximately 100 min/week at 4 months while those with PA levels ≥250 min/week at baseline reduced PA by an average of 33 min/week. This suggests that self-regulation approaches for weight gain prevention used in the current study can effectively increase PA among those with lower levels of PA at baseline; however additional intervention strategies may be needed within the context of weight gain prevention programs in order to promote maintenance of PA in those with high baseline levels. This is an important and interesting area of research which warrants further investigation, particularly given that PA is typically reduced throughout young adulthood, and our findings which demonstrated that a reduction in PA between months 4 and 24 was associated with weight gain. Novel strategies for promoting the maintenance of PA in this population should be explored given that the PA prescriptions used in the current study led to small reductions in PA among highly active individuals.

This study answers a novel research question related to weight gain prevention in young adults and has numerous strengths including a large sample size and unique population. It is further strengthened by the fact that it was addressed within a randomized trial, it included long-term follow-up data, and PA was assessed objectively and not via self-report measures. However, it is not without limitations. It is possible that the findings from this study would not be generalizable to the entire young adult population given that study participants may have been more motivated or health conscious, contributing to their decision to enroll in a weight gain prevention trial. Further, study participants were highly active, and predominately female and white. Finally, the findings highlighting the relationship between PA and weight change were performed post-hoc and thus future studies should be designed to examine the optimal dose of PA for weight gain prevention.

## Conclusions

In conclusion, this study suggests that both Small Changes (prescribed to increase steps by 2000 steps/day) and Large Changes (prescribed to increase bout-related MVPA to ≥250 min/week) PA recommendations led to similar increases in bout-related MVPA and daily steps, yet these changes were no different than those observed in the Self-guided group. This suggests that a brief intervention with general PA recommendations, as provided to Self-guided participants, may be sufficient for increasing PA at least temporarily among a group of active young adults who are concerned about weight gain. Moreover, study findings indicate that regardless of group assessment, better weight gain prevention over 2 years is associated with higher levels of bout-related MVPA, but not daily steps. Therefore, future weight gain prevention interventions should consider targeting bout-related MVPA and not daily steps.

## Figures and Tables

**Fig. 2:**
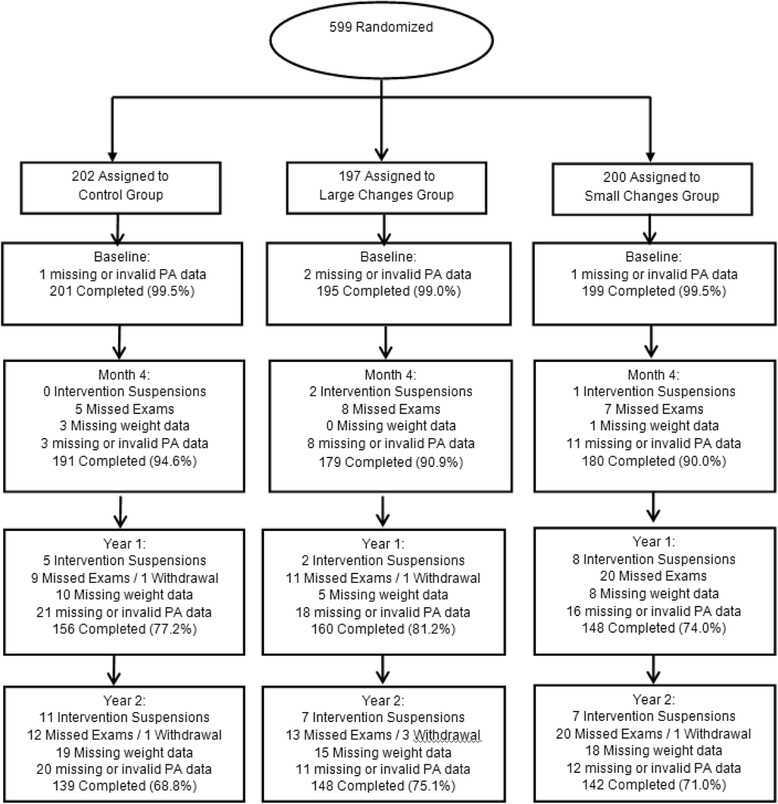
Flow of participants through the study

## Additional file

Additional file 1: CONSORT 2010 checklist of information to include when reporting a randomised trial*. (PDF 76 kb)
